# Effects of *Helicobacter pylori* eradication on esophageal motility, esophageal acid exposure, and gastroesophageal reflux disease symptoms

**DOI:** 10.3389/fcimb.2023.1082620

**Published:** 2023-03-08

**Authors:** Tong Zhao, Fang Liu, Yongjun Li

**Affiliations:** ^1^Department of Gastroenterology, Shihezi University School of Medicine, Shihezi, China; ^2^Department of Gastroenterology, First Affiliated Hospital, Shihezi University School of Medicine, Shihezi, China

**Keywords:** *Helicobacter pylori*, infection, eradication, gastroesophageal reflux disease (GERD), high-resolution esophageal manometry, 24-h esophageal pH monitoring

## Abstract

**Background:**

The effects of *Helicobacter pylori* (HP) eradication on gastroesophageal reflux disease (GERD) are yet to be fully elucidated. Few studies have investigated the mechanisms underlying the correlations between HP and GERD with prospective methods. The objective of this prospective clinical study was to explore the effects of HP eradication on GERD.

**Methods:**

Patients diagnosed with both GERD and HP were included. High-resolution esophageal manometry (HRM), 24-h esophageal pH monitoring, and the Gastroesophageal Reflux Disease Questionnaire (GerdQ) were performed before and after the successful eradication of HP, and the data were compared using statistical analysis.

**Results:**

Sixty-eight patients diagnosed with both GERD and HP were included. The After HP eradication group showed significantly decreased median distal contractile integral (DCI) [610.40 (847.45) vs. 444.90 (559.60)] and significantly increased median inefficient esophageal motility (IEM) [36.00 (50.00) vs. 60.00 (57.00)] in the HRM compared with those of the Before HP eradication group, indicating that HP eradication reduced esophageal peristalsis. The 24-h esophageal pH monitoring showed that the longest reflux event, the percentage of time that the pH was <4, the number of reflux episodes, and the DeMeester score were all significantly different between the Before and After HP eradication groups (P < 0.05), suggesting that HP eradication increased esophageal acid exposure. The After HP eradication group also had a significantly higher GerdQ score than that of the Before HP eradication group (P < 0.05).

**Conclusions:**

HP eradication reduced esophageal peristalsis, enhanced esophageal acid exposure, and aggravated GERD symptoms, suggesting that HP infection may be a protective factor for GERD.

## 1 Introduction

Gastroesophageal reflux disease (GERD) is a prevalent upper digestive tract disease, which primarily leads to acid reflux, dysphagia, heartburn, asthma, cough, and chest pain due to reflux of the contents from the stomach and duodenum into the esophagus (Vakil et al., 2006; Ashktorab et al., 2012; Maret-Ouda et al., 2020). GERD affects approximately 20% of the adult population in high-income countries (Maret-Ouda et al., 2020). The estimated prevalence of GERD is 13.3% of the population worldwide. There is a high incidence rate of 19.55% in North America, and a rising trend is also observed in the Asia-Pacific region (Fock et al., 2016; Eusebi et al., 2018). According to an epidemiological survey in 2020, the prevalence of GERD in China was 4.16% (Nirwan et al., 2020). The risk factors for GERD include female gender, smoking, genetic predisposition, nonsteroidal anti-inflammatory drug (NSAID) and aspirin use, and obesity (Eusebi et al., 2018; Maret-Ouda et al., 2020). Although GERD can be diagnosed based on the empirical proton pump inhibitor (PPI) therapy test and the presence of typical symptoms, additional diagnostic evaluation, such as ambulatory pH monitoring, high-resolution esophageal manometry (HRM), and digestive endoscopy, may also be required most of the time (Katz et al., 2013). The anti-reflux barrier of the esophagus includes the angle of His, the lower esophageal sphincter (LES), and the muscular fibers of the diaphragm. In most cases, there is a balance between the erosive effects of the reflux on the esophageal mucosa and the anti-reflux barrier of the esophagus. An impaired anti-reflux barrier and a weakened esophageal clearance function contribute to the occurrence of GERD (Satta et al., 2017).

*Helicobacter pylori* (HP) is the primary gastroduodenal pathogen related to the pathogenesis of gastritis, gastric carcinoma, and gastroduodenal ulcer. Early HP eradication reduces the occurrence of gastroduodenal ulcer and carcinoma. However, there is no consensus on the effects of HP eradication on GERD, and the mechanisms are still not entirely known. There are no unified standards for the application of anti-HP therapy in GERD patients. Several complications may occur after HP eradication, including GERD (Hojo et al., 2021). Some researchers have claimed a negative correlation between HP eradication and GERD or its typical symptoms (Moayyedi et al., 2001; Ashktorab et al., 2012) due to impaired gastric acid secretion. However, eradication of HP is recommended in the guidelines of the Italian Society of Gastroenterology and guidelines of Japan (Kato et al., 2019; Romano et al., 2022). Other explanations show that HP eradication has a beneficial effect on GERD (Miwa et al., 2002). A study in Japan showed an improvement of GERD symptom-related quality of life after HP eradication (Hirata et al., 2013). Additionally, some evidence suggested no connection between HP and GERD (Qian et al., 2011; Bor et al., 2017). Studies in different geographic regions may lead to entirely different outcomes. The results of HP eradication also depend on the form of gastritis in the patients with GERD (Yucel, 2019). Thus, the management of HP eradication in patients with GERD is controversial.

HRM measures the pressure from the pharynx to the stomach and therefore was used for the diagnosis of functional esophageal diseases in the 1990s (Pandolfino et al., 2009). HRM can also be used for localization of the LES, measurement of the esophageal pressure, accurate placement of the ambulatory pH monitoring catheters, and detection of the esophageal motor function before anti-reflux surgery (Gyawali et al., 2018; Patel et al., 2018). The 24-h esophageal pH (24-h pH) monitoring is a dynamic assessment of gastroesophageal reflux that allows an objective evaluation of acid reflux events and association with symptoms. The Gastroesophageal Reflux Disease Questionnaire (GerdQ) is a self-administered 6-item questionnaire to evaluate symptoms.

In this study, we collected and analyzed the results of HRM, 24-h pH, and endoscopic examination, as well as the GerdQ of patients diagnosed with both GERD and HP. We investigated the underlying mechanisms in order to elucidate the effects of HP eradication on GERD.

## 2 Materials and methods

### 2.1 Patients

A total of 234 patients who were diagnosed with both GERD and HP at the First Affiliated Hospital of Shihezi University between July 2021 and July 2022 were included in the study. The inclusion criteria were as follows: 1) diagnosed with GERD by both endoscopy and PPI test; 2) also diagnosed with HP infection by biopsy examination or ^14^C-urea breath test (^14^C-UBT); 3) willingness to undergo HP eradication therapy and HRM and 24-h pH; and 4) aged from 18 to 80 years. Patients were excluded if they had hiatal hernia, underwent gastric or esophageal surgery, or consumed food that could have affected the gastrointestinal motor function or acid reflux before the study. Patients with a history of acid secretion inhibitor and gastrointestinal motility drug usage in the 1 week prior to the study were also excluded. All patients signed an informed consent, and the study was approved by the local ethics committee.

All participants were treated with PPI-based quadruple therapy (colloidal bismuth pectin, 600 mg, thrice daily; omeprazole, 40 mg, once daily; amoxycillin, 100 mg, twice daily; clarithromycin, 50 mg, twice daily) for 14 days. One month after the PPI-based quadruple therapy, patients underwent the ^14^C-UBT. After successful HP eradication, all patients performed a second GerdQ, HRM, and 24-h pH.

### 2.2 Helicobacter pylori test

During upper gastrointestinal endoscopy, the corpus and antrum tissue specimens were obtained and fixed in formalin. Biopsy specimens were subjected to the rapid urease test (RUT). HP infection was also determined by the ^14^C-UBT (Thor and Błaut, 2006). Being positive in either the RUT or the ^14^C-UBT suggested HP infection.

### 2.3 Upper gastrointestinal endoscopy

The endoscopic images of the reflux esophagitis (RE) were categorized based on the confluence of erosion and the longest length of the mucosal break according to the Los Angeles classification. RE was graded from A (the lowest severity) to D (the highest severity). GERD also included Barrett’s esophagus and non-erosive reflux disease (NERD). Patients having the symptoms without endoscopic esophageal abnormalities were considered to have NERD, which can be evaluated by the functional esophageal test. All patients underwent upper gastrointestinal endoscopy. The endoscopic findings were judged by two experienced endoscopists separately. When the judgments were different, the final conclusions were unified by discussion.

### 2.4 Gastroesophageal reflux disease questionnaire (GerdQ)

**Figure 1:**
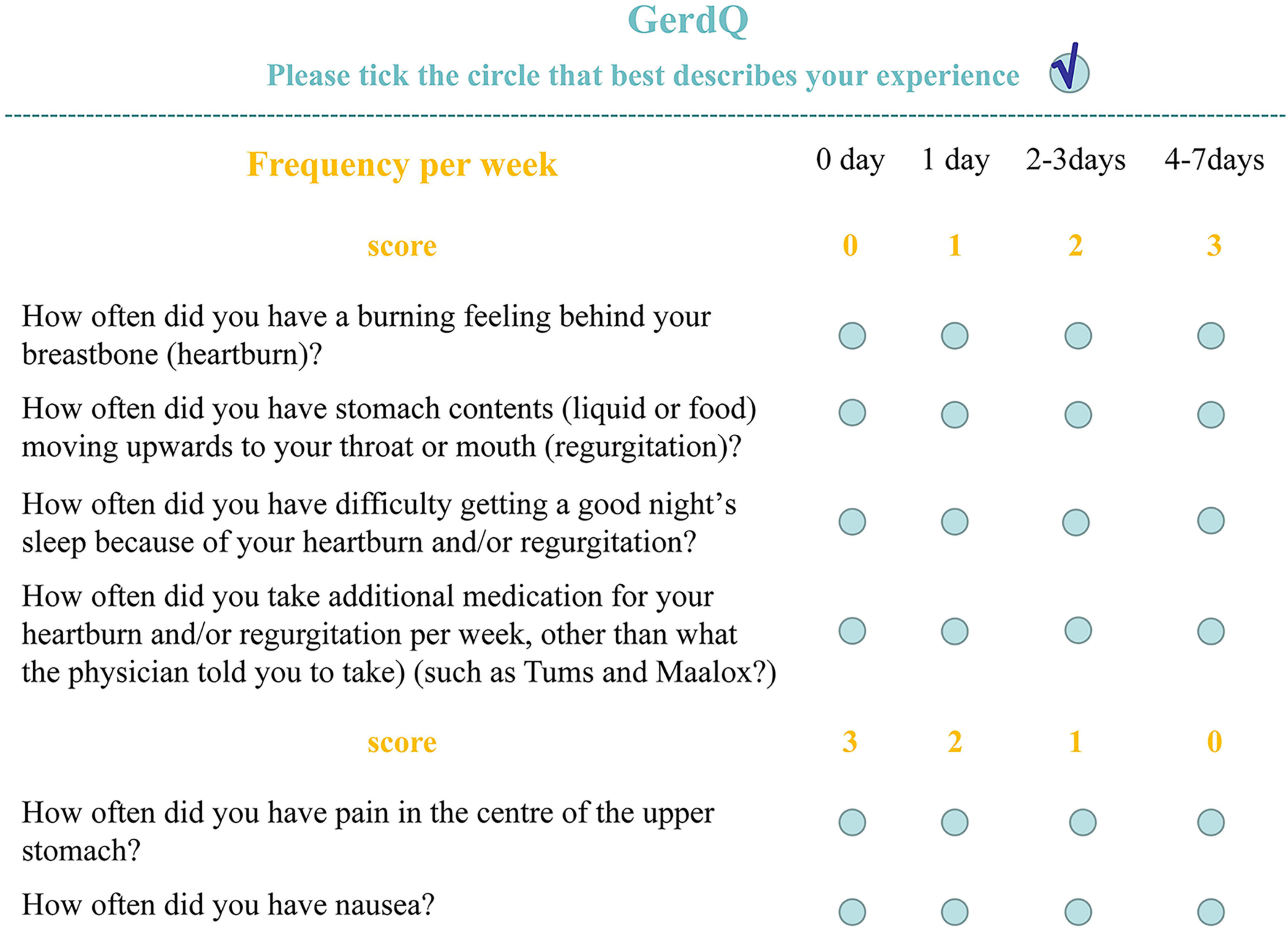
GerdQ score.

The GerdQ is used to assess reflux-related symptoms in the gastroenterology clinics. It covers six reflux-related symptoms, namely, heartburn, acid regurgitation, epigastric soreness, nausea, dyssomnia, and whether taking over-the-counter drugs. The GerdQ has a high diagnostic value for GERD (Bai et al., 2013). The GerdQ includes six questions and is shown in **Figure 1**. Patients with a total GerdQ score of over 8 have a higher possibility of developing GERD than patients with a score of 8 or below. The GerdQ was administered to all recruited patients by a research assistant prior to endoscopy.

### 2.5 High resolution esophageal manometry (HRM)

HRM is commonly considered the gold standard for detecting motility disorders and anatomic associations at the esophagogastric junction. It shows the characteristics of the resting esophageal sphincter and the esophageal motor function during swallowing. The Chicago Classification (CC) is a classification scheme that allows the diagnosis of GERD based on manometry. CC v4.0 is the updated version (Fox et al., 2021; Yadlapati et al., 2021).

**Figure 2:**
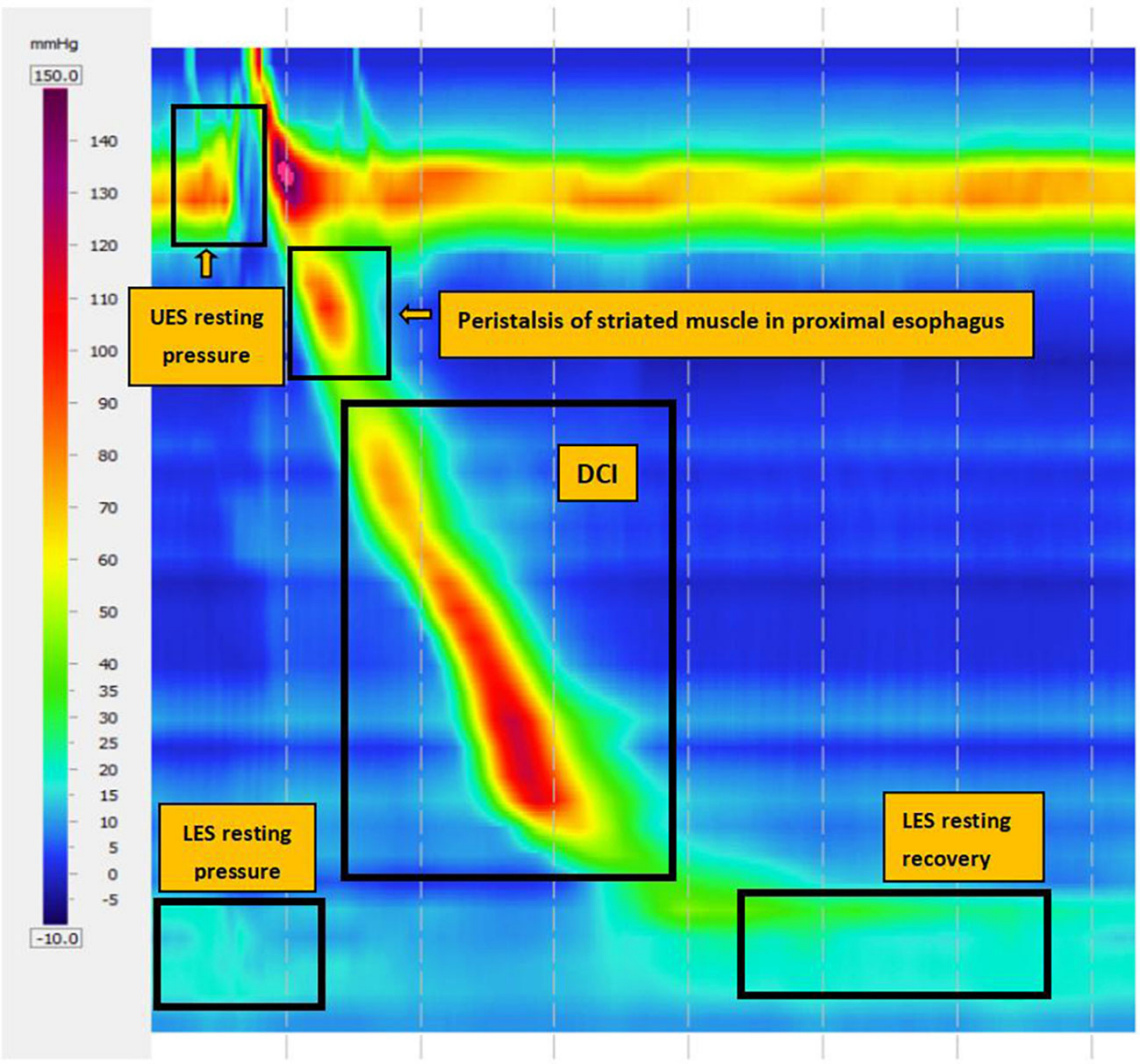
High resolution esophageal manometry (HRM) images.

The main metrics in the HRM are the integrated relaxation pressure (IRP), which measures deglutitive relaxation across the LES, and distal contractile integral (DCI), which detects the comprehensive value of length, pressure, and duration of esophageal contraction to evaluate the strength of esophageal body contraction. In CC v4.0, inefficient esophageal motility (IEM) is defined as normal IRP with 50% failed peristalsis or more than 70% ineffective swallows. Major peristalsis breaks (PBs) are defined as PBs longer than 5 cm for measuring with or without esophageal PBs (**Figure 2**).

### 2.6 24-h esophageal pH monitoring

Ambulatory pH monitoring, which provides objective measures of acid reflux events and symptoms, is used to diagnose the reflux of esophageal gastric acid. All participants followed a restricted diet and consumed no medication or food that might affect the results. The parameters assessed during the 24-h pH monitoring included the number of reflux episodes, the number of reflux events >5 min, the acid exposure time percentile (AET%), the longest reflux event, and the DeMeester score. The AET% was defined as the percentage of the total time that the pH was <4, and when AET% >6%, the data were considered abnormal. A DeMeester score >14.72 was considered to indicate pathologic acid reflux (Gyawali et al., 2020).

### 2.7 Statistical analysis

The SPSS software (version 22; SPSS Inc., Chicago, IL, USA) was used for data analysis. Continuous variables [age, body mass index (BMI), HRM parameters, 24-h pH monitoring parameters, and GerdQ scores] were expressed as mean ± standard deviation (mean ± SD) when data followed a normal distribution. When not obeying a normal distribution, they were expressed as median (quartile) or median [interquartile range (IQR)]. Categorical variables (gender, smoking, and drinking status) were presented as numbers and percentages. The differences between before and after HP eradication were calculated. When differences between paired data followed a normal distribution, a paired t-test was performed. When differences between paired data followed a skewed distribution, a nonparametric Wilcoxon rank sum test was performed to compare them. A P-value <0.05 was considered significant.

To analyze the risk factors, univariate conditional logistic regression models were first used. We calculated 95% confidence intervals (CIs) and odds ratios (ORs) to evaluate the strengths of the correlations. Clinically plausible variables identified in the univariate analysis were included in a multivariable conditional logistic regression model in a stepwise selection manner if P < 0.05.

## 3 Results

### 3.1 Basic characteristics of patients

**Figure 3:**
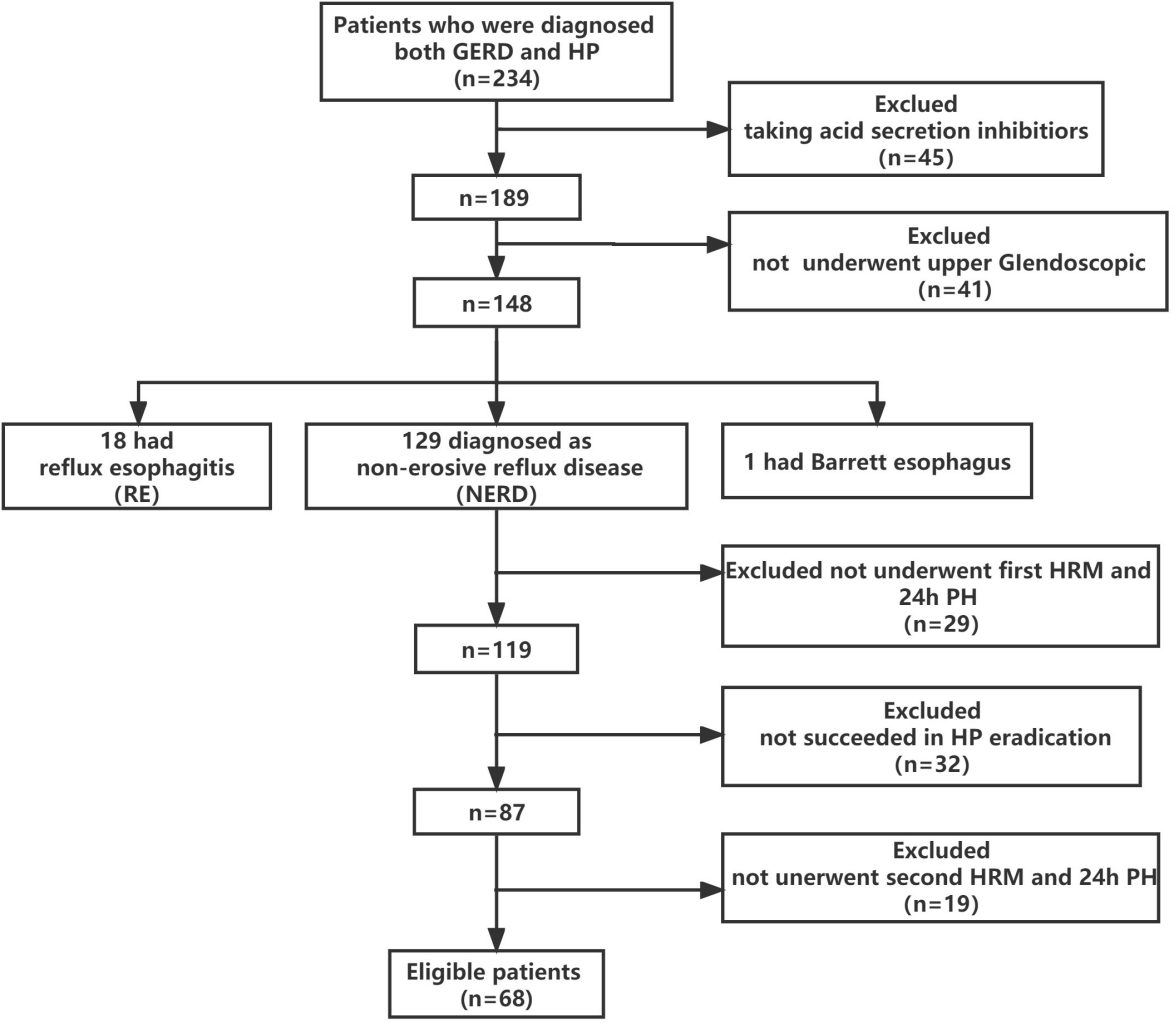
Recruitment flowchart. A total of 234 patients were screened, and 166 were excluded due to 1) taking H2-receptor antagonists or PPIs (n = 45), 2) not undergoing upper GI endoscopy (n = 41), 3) unsuccessful first HRM and 24-h pH monitoring (n = 29), 4) unsuccessful HP eradication (n = 32), and unsuccessful second HRM and 24-h pH monitoring (n = 19).

Sixty-eight patients who underwent successful HP eradication therapy and completed the esophageal function examination before and after the therapy were recruited (**Figure 3**). There were 18 patients with RE, 129 patients with NERD, and one patient with Barrett’s esophagus. There were 46 men and 22 women (mean age = 52.06 years) (**Table 1**). The risk factors for GERD were identified by univariate and multivariate analyses. Results showed that three potential risk factors were associated with GERD, namely, high BMI and smoking and drinking habits (**Table 2**).

**Table 1:** Patient characteristics.

	Diagnosed with both GERD and *H. pylori*
Numbers	68
Age(Mean ± SD), Median(IQR)	52.06 ± 11.99, 52.50(47-59)
Gender, Female, n(%)	22(32.00)
BMI (kg/m^2^) (Mean ± SD), Median(IQR)	22.85 ± 1.47, 22.84(21.51-23.84)
Smoker, Yes, n(%)	38(55.88)
Drinker, Yes, n(%)	36(52.94)

**Table 2:** Univariate and multivariate analyses of the risk factors for GERD.

Characteristics	n=68	Univariate		Multivariate	
		P	OR (95%CI)	P	OR (95%CI)
Gender, Female n (%)	22 (32.00)	0.092	2.685 (0.966-7.463)		
Smoking, Absent n (%)	38 (55.88)	0.019	4.552 (1.448-14.921)	0.031	6.959 (1.194-40.564)
Drinking, Absent n (%)	36 (52.94)	0.034	3.500 (1.220-10.004)	0.038	7.582 (1.124-51.163)
BMI (Mean ± SD)	22.85 ± 1.47	0.002	4.190 (1.420-12.369)	0.003	7.495 (1.973-28.466)
Age (Mean ± SD)	52.06 ± 11.99	0.303	1.030 (0.982-1.080)		

### 3.2 HRM parameters

**Figure 4:**
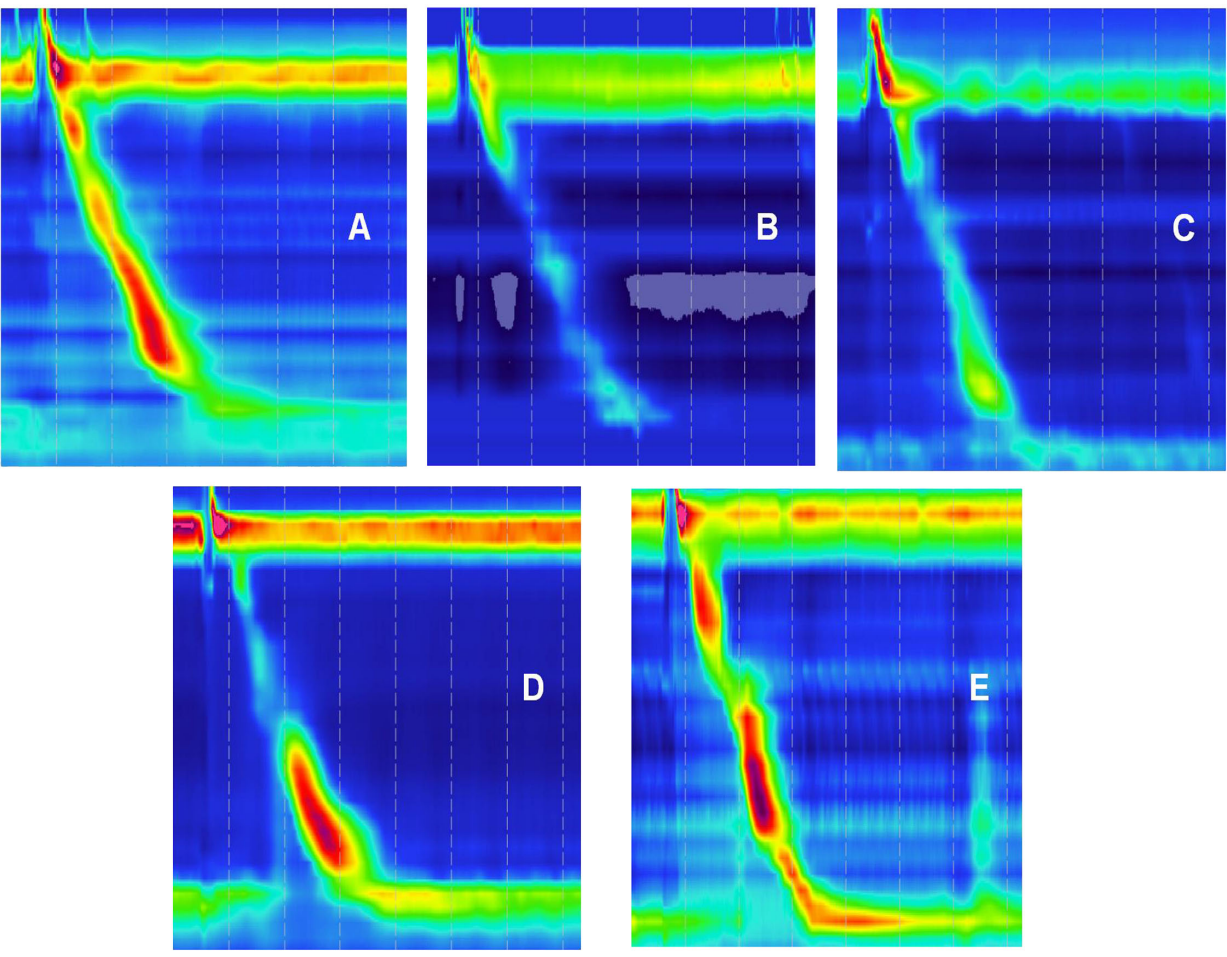
Highresolution esophageal manometry (HRM) images. **(A)** Normal HRM image. **(B–E)** Abnormal HRM.

Normal and abnormal HRM images are shown in **Figure 4**. The After HP eradication group showed a significantly decreased median DCI [610.40 (847.45) vs. 444.90 (559.60)] and a significantly increased median IEM [36.00 (50.00) vs. 60.00 (57.00)] in the HRM compared to those of the Before HP eradication group (P < 0.05), indicating that HP eradication reduced esophageal peristalsis. There was no significant difference in the IRP, LES pressure, and PB >5 cm rate between the two groups, indicating that HP eradication cannot change the LES pressure and large PBs (**Table 3**).

**Table 3:** HRM parameters before and after HP eradication.

	Before HP eradication	After HP eradication	P
LES pressure (mmHg)	6.27 ± 4.59	6.41 ± 4.95	0.875
IRP (mmHg)	5.58 ± 4.33	5.40 (6.35)	0.875
PB >5 cm rate (%)	0.00 (0.00)	0.00 (9.00)	0.161
DCI (mmHg·s·cm)	610.40 (847.45)	444.90 (559.60)	0.001
IEM (%)	36.00 (50.00)	60.00 (57.00)	0.000

Statistical comparison: paired-sample t-test: LES pressure, IRP, and IEM. Nonparametric Wilcoxon rank sum test: PB >5 cm and DCI. Data are expressed as mean ± SD or median (quartile).

### 3.3 24-h esophageal pH monitoring

The 24-h pH showed that the longest reflux event, esophageal acid exposure time (AET%), number of reflux episodes, and DeMeester score were statistically significant between the Before and After HP eradication groups (P < 0.05) (**Table 4**). Our results showed that HP eradication increased the esophageal acid exposure and reflux and damaged the anti-reflux barrier. HP seems to have a protective role in GERD patients.

**Table 4:** The 24-h esophageal pH monitoring before and after HP eradication.

	Before HP eradication	After HP eradication	P
AET (%)	23.64 ± 18.49	37.35 ± 26.89	0.008
Number of reflux episodes	113.97 ± 84.87	203.00 (64.00)	0.008
DeMeester score	49.00 (141.90)	71.40 (233.85)	0.008
Reflux events >5 min	27.00 (33.00)	45.00 (110.00)	0.085
Longest reflux event	6.00 (12.50)	17.78 ± 11.67	0.011

Statistical comparison: paired-sample t-test: Reflux events >5 min. Nonparametric Wilcoxon rank sum test: AET, number of reflux episodes, DeMeester score, and longest reflux event. Data are expressed as mean ± SD or median (quartile).

### 3.4 GerdQ score

The GerdQ score of the After HP eradication group was significantly lower than that of the Before HP eradication group (P < 0.05), suggesting that patients after HP eradication had more severe symptoms including heartburn, acid regurgitation, epigastric soreness, nausea, dyssomnia, and whether taking over-the-counter drugs (**Table 5**).

**Table 5:** The GerdQ score before and after HP eradication.

	Before HP eradication	After HP eradication	P
GerdQ score	11.00 (2.00)	12.00 (3.00)	0.030

Statistical comparison: nonparametric Wilcoxon rank sum test: GerdQ score. Data are expressed as median (quartile).

## 4 Discussion

The role of HP eradication in GERD pathogenesis remains controversial, and the mechanisms are not yet fully understood. There are no unified criteria for the application of anti-HP therapy in GERD patients. Some researchers have claimed that HP eradication leads to GERD (Xie et al., 2013; Hojo et al., 2021), while another explanation has shown that HP eradication has a beneficial effect on GERD (Hirata et al., 2013). Additionally, some have suggested no correlation between HP and GERD (Bor et al., 2017). There are several possible mechanisms of HP eradication leading to GERD. One of the mechanisms is that HP infection increases the acid reflux owing to the disappearance of neutralization of bacterial ammonia (Arents et al., 2001; Queiroz et al., 2004). One hypothesis for the protective mechanism is that HP infection results in atrophy of the gastric mucosa and damage of acid production (Hirata et al., 2013). The nitric oxide synthase (NOS) regulation system is a modulator of the inflammatory reaction in the gastric mucosa of HP that can induce NO release to inhibit gastric acid secretion (Slomiany and Slomiany, 2011). Another possible explanation is that HP infection makes the vagus nerve receptor on the gastric fundus and cardia active, which enhances the secretion of serum gastrin and increases the LES pressure, reduces the reflux of gastric contents, and protects the esophageal mucosa (Thor and Błaut, 2006). Some researchers have claimed that the protective mechanism of HP is regarded as its negative impact on ghrelin and gastric acid production, and stomach ghrelin stimulates appetite, leading to obesity, which is a widely known risk factor in the development of GERD (Goll et al., 2007; Rubenstein et al., 2013). Others have hypothesized that HP eradication has a beneficial effect on GERD. In some patients with HP, the organism colonizes the antrum preferentially, resulting in an antrum-dominant gastritis characterized by aggravated GERD symptoms and increased gastrin and acid secretion. HP eradication reduced acid secretion (El-Omar et al., 1995; Vicari et al., 1998; Zullo et al., 2013).

In patients with GERD on PPI therapy, eradication of HP is recommended in the guidelines of the Italian Society of Gastroenterology and guidelines of Japan (Kato et al., 2019; Romano et al., 2022). However, the guidelines of the American College of Gastroenterology (ACG) in 2013 indicated that screening for HP infection is not recommended in GERD patients, and treatment of HP infection is not routinely required as a part of the anti-reflux therapy (Katz et al., 2013). This recommendation was not mentioned in the ACG 2022 guidelines (Katz et al., 2022).Our results showed that HP eradication aggravated GERD. However, early HP eradication reduces the occurrence of gastroduodenal ulcer and carcinoma. Thus, HP eradication should proceed with uncertainty.

IEM is a highly etiologically diagnosed type of esophageal dynamic disorder (Gyawali et al., 2019). DCI is used to evaluate the strength of esophageal body contraction. In this study, patients after HP eradication showed significantly increased IEM and reduced DCI, indicating that HP eradication reduced esophageal peristalsis. A majority of parameters measured in the 24-h pH monitoring in patients after HP eradication were significantly higher than those in patients before HP eradication (P < 0.05), suggesting that patients treated with anti-HP therapy had higher acid exposure in the esophagus. Our results support the hypothesis that HP eradication increases gastric acid production and reflux. However, our data do not support the viewpoint that HP eradication changes LES pressure. There was no statistical significance in reflux events >5 min between the two groups possibly owing to the small sample size. The GerdQ score also increased in the second examination, indicating that patients showed more severe symptoms after HP eradication, further showing a negative correlation between HP eradication and GERD symptoms.

The results of GERD after HP eradication are most likely to depend on the form of gastritis (antrum-predominant active or corpus-predominant active). In western countries, antrum-predominant gastritis is the most common type in patients with GERD and prevalent in children and young adults. In Asia, corpus-predominant and atrophic gastritis is more frequent, and it appears that patients with HP infection have impaired acid secretion. After HP eradication, a repaired corpus mucosa and the recovery of acid secretion may promote the development of GERD (Haruma, 2004; Naylor et al., 2006).

Bacterial virulence is important in determining acid secretion. The cytotoxin-associated gene (Cag) protein can inhibit cytokine production such as interleukin 1, which probably reduces gastric acid. In addition, the vacuolating cytotoxin A (VacA), especially the s1m1, reduces gastric acid secretion by damaging the gastric parietal cells, which may be a protective mechanism against GERD (Yucel, 2019). According to previous studies, CagA-positive HP strains may play a protective role in the development of GERD, especially in East Asian countries (Azuma et al., 2004; Ashktorab et al., 2012; Chiba et al., 2012). It has been reported that no association was detected between CagE HP strains and GERD (Godoy et al., 2003). An Iranian study showed that there was no difference between GERD patients and controls in the prevalence of HP, but the presence of the CagA strains and the coexistence of CagA and CagE strains were higher in the control group (Shavalipour et al., 2017).

In this study, one of the limitations is the insufficient sample size. Many patients refused to undergo the functional examinations due to nausea caused by catheterization. Patients underwent unsuccessful HP eradication owing to the failure to adhere to the medication regimen. Other limitations are the lack of information on the virulence of the clinical strains responsible for infection, the composition of the microbiota, and the type of the different patients enrolled. The strengths of this study are as follows: 1) Instead of performing a retrospective study, we prospectively collected and compared the results of the same cohort before and after HP eradication. 2) Both the 24-h pH monitoring and HRM were performed, aiming to investigate not only the correlation between HP and GERD but also the underlying mechanisms with evidence. HP infection affected the reflux of acid. Our data indicated that it also affected esophageal motility, which should be further investigated.

## 5 Conclusion

This study showed that HP eradication therapy increased esophageal acid production and reflux, reduced esophageal peristalsis, and aggravated GERD symptoms in patients diagnosed with both HP infection and GERD, suggesting the protective role of HP in GERD. These findings may have implications for whether HP eradication should be used in clinical practice. More investigations are required to further explore the effects of HP on GERD patients.

## Data availability statement

The original contributions presented in the study are included in the article/supplementary material. Further inquiries can be directed to the corresponding author.

## Ethics statement

The studies involving human participants were reviewed and approved by the Ethics committee of first Affiliated Hospital, Shihezi University School of Medicine (protocol no.KJX-2021-051-02). The patients/participants provided their written informed consent to participate in this study. Written informed consent has been obtained from the patients to publish this paper.

## Author contributions

TZ made a substantial contribution to the concept or design of the work; TZ and FL made a contribution to acquisition, analysis and interpretation of data; TZ drafted the article; YL revised the article critically for important intellectual content. All authors contributed to the article and approved the submitted version.
